# Supplementary material for: Unequal mitochondrial segregation promotes asymmetric fates during neurogenesis
Source: Nat Commun. 2025 Dec 15;16:11049. doi: 10.1038/s41467-025-66932-0 (PMC12706016; doi:10.1038/s41467-025-66932-0)
Supplement: Supplementary file 1 — Supplementary Information [file 41467_2025_66932_MOESM1_ESM.pdf]

Supplementary information

Unequal mitochondrial segregation promotes asymmetric fates during neurogenesis

Benjamin Bunel [1,2], Rémi Leclercq [1], Rosette Goiame [1], Arnaud Gautier [3,4], Xavier Morin [5*†], Evelyne Fischer [6*†]

[1] Institut de Biologie de l'Ecole Normale Supérieure (IBENS), CNRS, Inserm, Ecole Normale Supérieure, PSL Research University, 75005 Paris, France.

[2] Sorbonne Université, Collège Doctoral, 75005 Paris, France.

[3] Sorbonne Université, École Normale Supérieure, Université PSL, CNRS, Laboratoire des Biomolécules, 75005 Paris, France.

[4] Institut Universitaire de France, 75005 Paris, France.

[5] Institut de Biologie de l'Ecole Normale Supérieure (IBENS), CNRS, Inserm, Ecole Normale Supérieure, PSL Research University, 75005 Paris, France. Electronic address: xavier.morin@ens.fr

[6] Institut de Biologie de l'Ecole Normale Supérieure (IBENS), CNRS, Inserm, Ecole Normale Supérieure, PSL Research University, 75005 Paris, France. Electronic address: evelyne.fischer@ens.fr

* : co-corresponding authors

† : these authors contributed equally to this work

This document contains 9 Supplementary Figures, one Supplementary Table, and supplementary references.

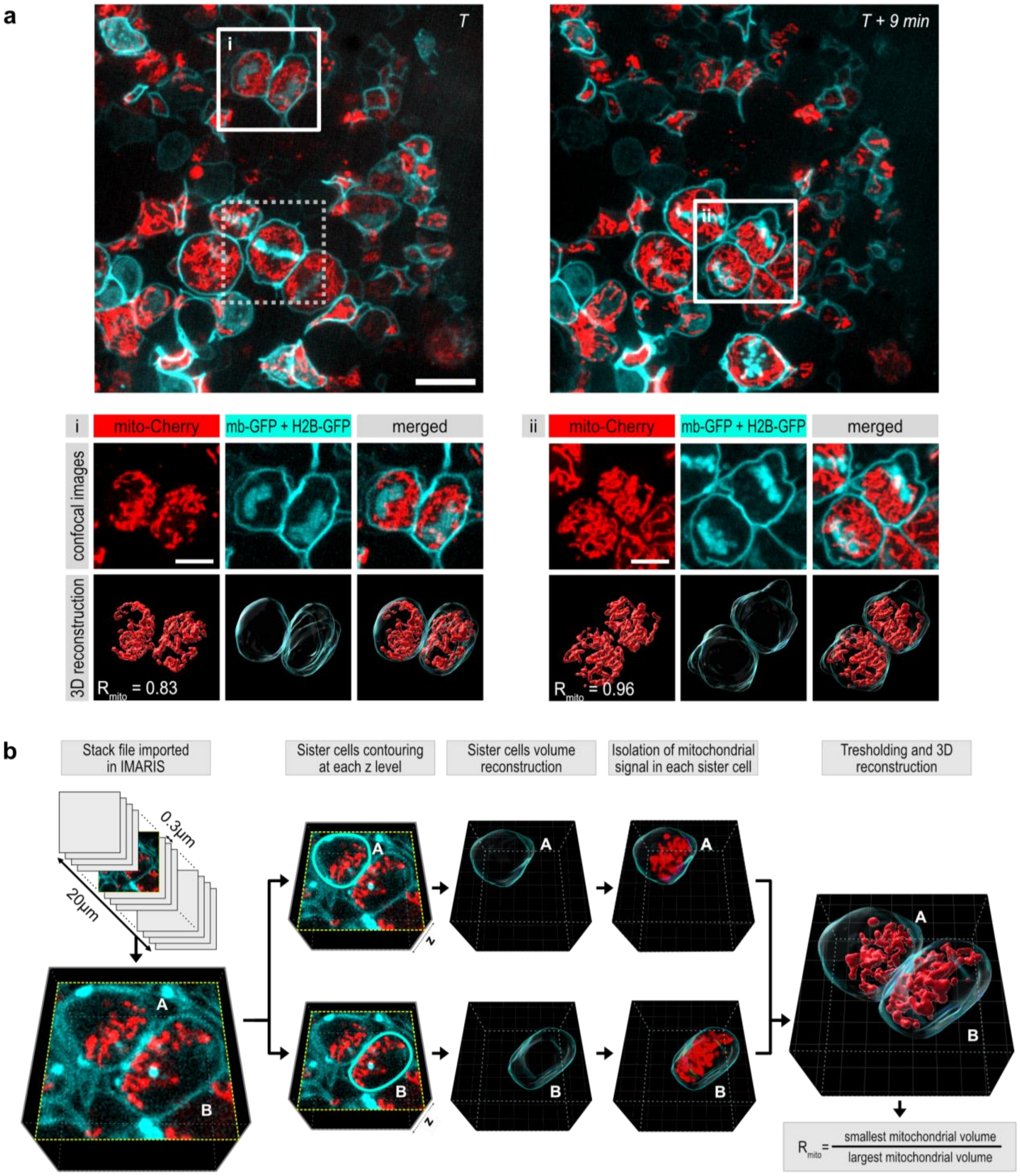

Supplementary Fig.1. 3D reconstruction of mitochondrial volume in daughter cells from a neural progenitor. Related to Fig.1.

a. Top panels: en-face view of a single z-plane from a 90*90µm field of a neural tube in live imaging at two different time points. Red : Mitochondria, Cyan : cell contour.

Bottom panels show images and 3D reconstructions of mitochondria in pairs of daughter cells of two progenitors dividing at successive timepoints at E2. The cells in i) and ii) correspond to white boxes in upper left and upper right panels, respectively). The dashed white

box on the upper left panel highlights a metaphase progenitor whose daughter cells are visible in panel ii. Upper rows in i) and ii): z plane of cell contour and chromosomes (middle, cyan), mitochondria (left, red), and merged (right) live fluorescence; Bottom rows: corresponding 3D reconstructed images from the entire z-stacks. Scale bars 5µm.

b. 3D reconstruction pipeline: Stack files of images of daughter cells immediately after division (~25µm deep z-stacks with a 0.3µm z-step) were imported in IMARIS software (Bitplane) to perform cell volume and mitochondrial volume 3D reconstructions. Each daughter cell volume was delineated manually by drawing cell contour on each z plane through their entire height using either the membrane reporter signal or a cytoplasmic reporter signal, and reconstructed using the surface creation tool of IMARIS. The mitochondrial signal contained in the cellular volume of each daughter cell was then isolated by masking mitochondrial signal located outside of this volume. Each daughter's mitochondrial volume was then reconstructed with the surface creation tool, using the same threshold for both cells. Values of mitochondrial volumes for each daughter cells were then used to calculate R_{mito}.

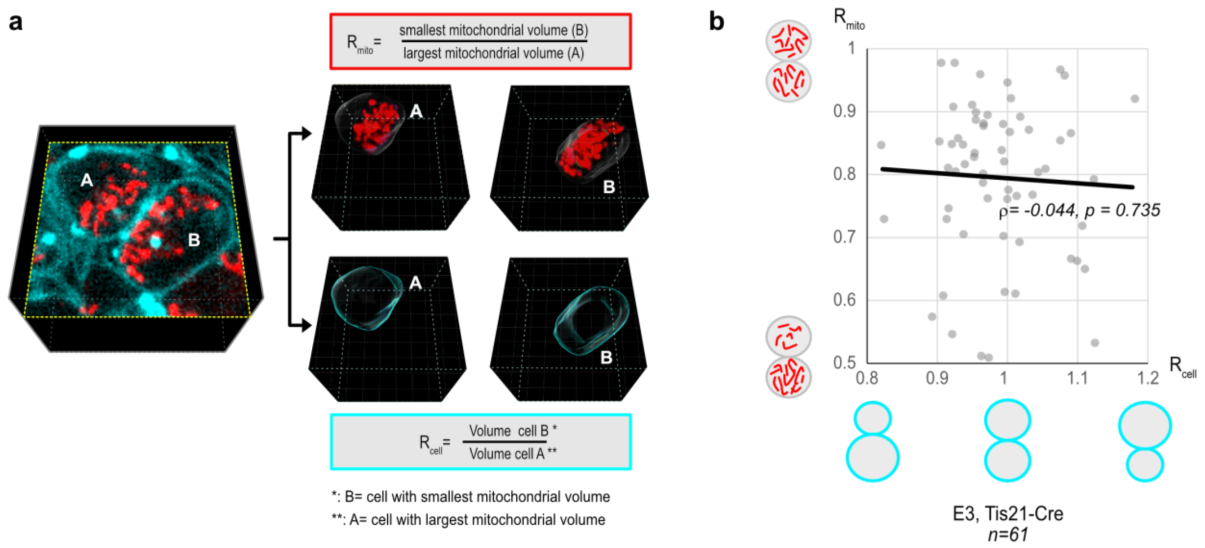

Supplementary Fig.2. Absence of correspondence between mitochondrial inheritance ratio and cell volume ratio. Related to Fig.1.

a. 3D reconstruction of cell volume in pairs of daughter cells born from a neural progenitor. Stack file of images of daughter cells immediately after division (~25µm deep z-stacks with a 0.3µm z-step) were imported in IMARIS software (Bitplane) to perform cell volume and mitochondrial volume 3D reconstructions. Each daughter cell volume was delineated manually by drawing cell contour on each z plane through their entire height using either the membrane reporter signal and reconstructed using the surface creation tool of IMARIS. The mitochondrial signal contained in the cellular volume of each daughter cell was then isolated by masking mitochondrial signal located outside of this volume. Each daughter's mitochondrial volume was then reconstructed with the surface creation tool, using the same threshold for both cells. Data of both mitochondrial and cellular volumes for each daughter cells were then exported from Imaris to perform ratio measurements to assess the mitochondrial distribution between daughter cells and to compare their cellular volume. Note that the directionality of the R_{cell} ratio is dictated by R_{mito}, and that as a consequence, R_{cell} values can be higher than 1.

b. Graph plotting the inherited mitochondrial volume ratio (R_{mito}, Y axis) and corresponding cell volume ratio (R_{cell}, X axis) of pairs of sister cells in the Tis21-Cre population at E3. Black line shows the calculated regression line between the two distributions. Spearman rank's correlation coefficient analysis revealed no significant correlation between the two variables ($\rho = -0.044$, $p = 0.735$). N=61 pairs of Tis21-Cre expressing cells from 7 embryos**.**

E = Embryonic day. Source data are provided as a Source Data file.

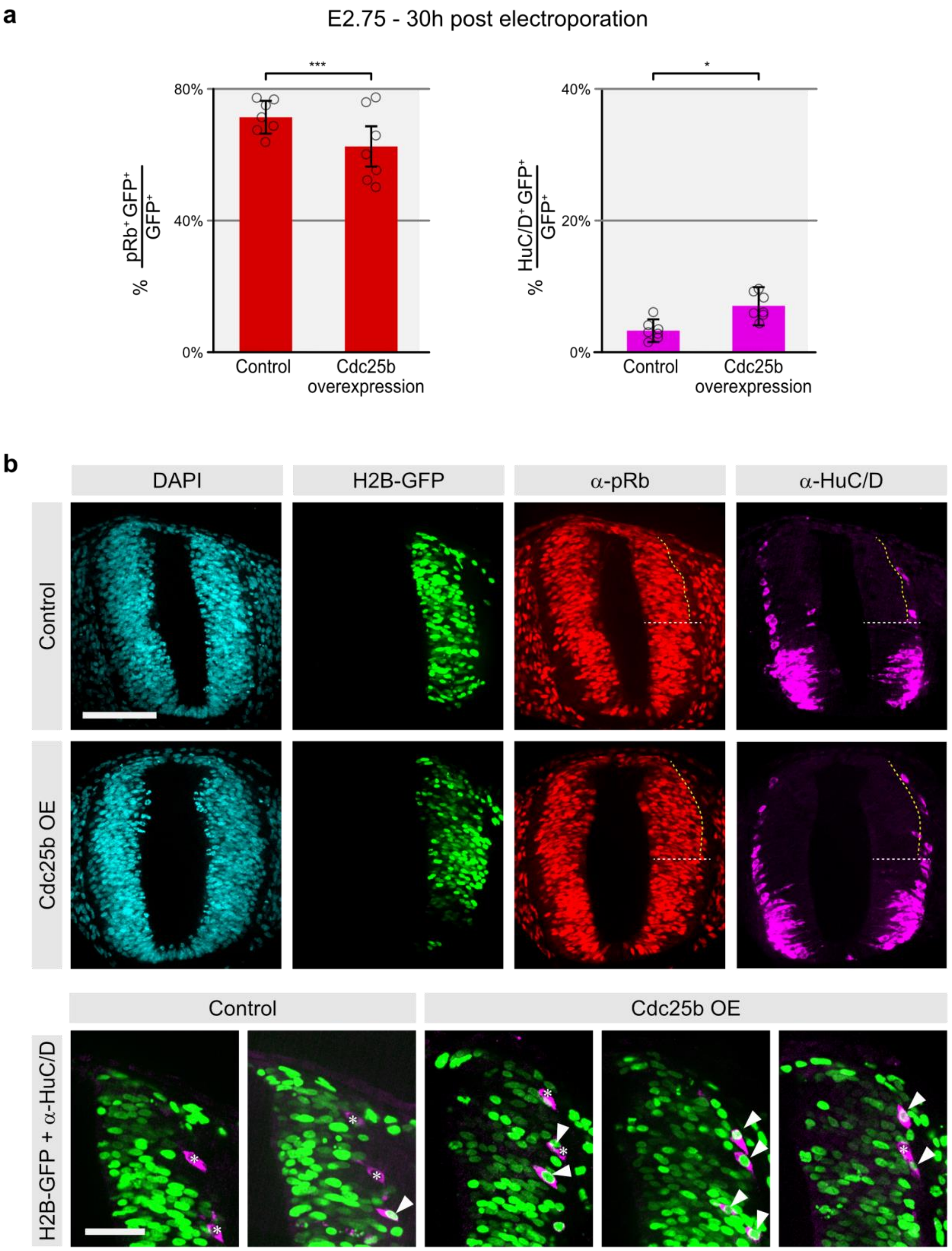

Supplementary Fig.3. Increased neurogenesis upon Cdc25b overexpression in the chick embryonic neural tube. Related to Fig.2.

a. Distribution of the pRb positive cells (left graph, red) and HuC/D positive neurons (right graph, magenta) in control or Cdc25b overexpression conditions at E2.75, 30 hours after

electroporation (30 hours post EP). CTRL shRNA: 1951 GFP positive cells from 7 embryos, Cdc25b shRNA: 1875 GFP positive cells from 7 embryos. Error bars = SEM. Statistical analysis was performed using two-tailed unpaired student's t-test. ***: $p = 0.0006$; *: $p = 0.0102$.

b. Top panels: representative transverse sections (4 µm z-projections) of the electroporated chick neural tube (thoracic level) at E2.75. Immunofluorescence with pRb antibody to label progenitors (red, nuclear signal) and with HuC/D antibody to label neurons (magenta, cytoplasmic signal) in control and overexpression of Cdc25b (Cdc25b OE) conditions. H2B-GFP fluorescence (green) represents the fluorescent electroporation reporter and DAPI staining is shown in cyan. The white horizontal dashed lines show the lower limit of the counting zone. The separation between ventricular and mantle zones in the counted region is marked by a yellow dashed line. Scale bar: 100µm

Bottom panels: representative close ups (4 µm z-projections) showing HuC/D positive electroporated cells (white arrows) and non-electroporated HuC/D positive cells (asterisks) in the control and overexpression conditions. Scale bar: 40µm.

E = Embryonic day; h = hours; OE = overexpression. Source data are provided as a Source Data file.

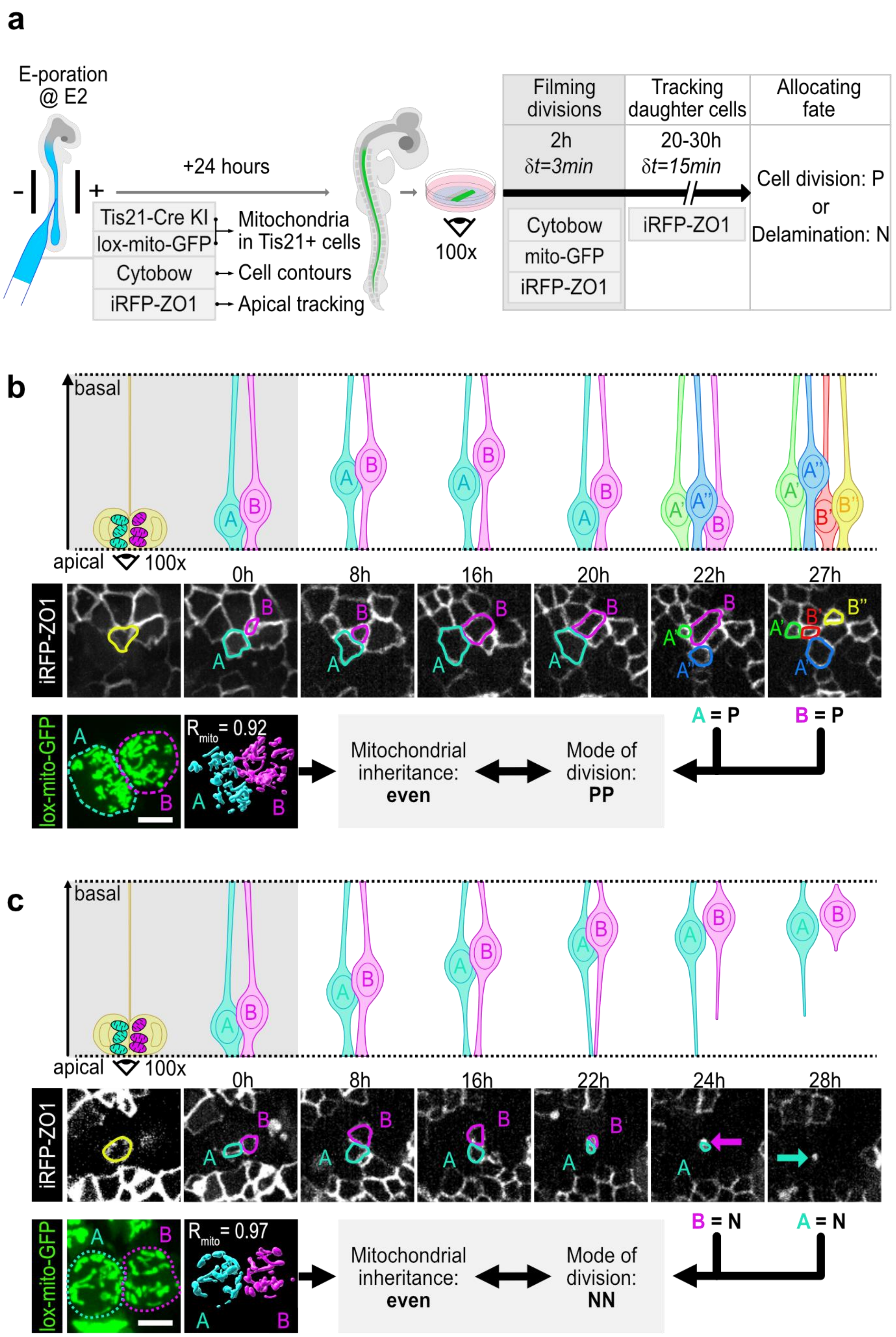

a
E-poration @ E2
+24 hours
Tis21-Cre KI
lox-mito-GFP
Cytobow
iRFP-ZO1
Mitochondria in Tis21+ cells
Cell contours
Apical tracking
100x
Filming divisions
Tracking daughter cells
Allocating fate
2h
δt=3min
20-30h
δt=15min
Cell division: P or Delamination: N
Cytobow
mito-GFP
iRFP-ZO1
iRFP-ZO1
b
basal
apical
100x
0h
8h
16h
20h
22h
27h
iRFP-ZO1
lox-mito-GFP
R mito = 0.92
A = P
B = P
Mitochondrial inheritance: even
Mode of division: PP
c
basal
apical
100x
0h
8h
16h
22h
24h
28h
iRFP-ZO1
lox-mito-GFP
R mito = 0.97
B = N
A = N
Mitochondrial inheritance: even
Mode of division: NN

Supplementary Fig.4. Fate tracking in pairs of daughter cells from a Tis21 positive progenitor showing even mitochondrial distribution and symmetric modes of division. Related to Fig.2.

a. Experimental strategy to combine live monitoring of mitotic mitochondrial inheritance and tracking of daughter cell fate. *In ovo* electroporation of a combination of five vectors carrying DNA constructs for Tis21-P2A-Cre somatic knock-in, and fluorescent reporters for mitochondria (lox-mito-GFP), apical tracking (iRFP-ZO1) and cytoplasm staining (Cytobow) in the chick embryonic neural tube at E2. Embryos were harvested at E3, and neural tubes were mounted for en-face imaging. The neuroepithelium was imaged at high spatio-temporal resolution (δt=3min and z=0.3µm) for two hours to record mitochondrial inheritance during progenitor divisions, followed by tracking of the daughter cells by imaging the apical surface at lower resolution (δt=15min and z=1µm, 20µm stack height centered on iRFP-ZO1 staining) for 20 to 30 hours.

b-c. Time-lapse series (en-face imaging) of dividing neural progenitors showing even mitochondrial distribution and symmetric PP (B) or NN (C) modes of division. Top rows: Schematic representation of the time course from the division of a progenitor to the determination of its daughter's fate based on their behavior and morphological criteria (new division for a progenitor, delamination for a neuron). Middle rows show time-lapse series (en-face imaging) of the iRFP-ZO1 signal used for long term tracking of pairs of daughter cells. In panel B, both daughter cells A and B are progenitors (as deduced from their division producing A' and A'', and B' and B''), while in panel C, both daughters are neurons (as deduced from the progressive reduction of their apical surface followed by delamination at 24 hours and 28 hours). Bottom rows: mitochondrial distribution (R_{mito}) is measured immediately after cytokinesis of the mother cell and matched with the mode of division deduced from the fate of the daughters. Scale bars in B-D: 5µm.

E = Embryonic day; E-poration = electroporation; P = progenitor; N = neuron; δt = interval between time-lapse frames; h = hours; min = minutes. Gray shading in the top parts of panels b and c represents the period of high temporal resolution imaging (δt = 3min).

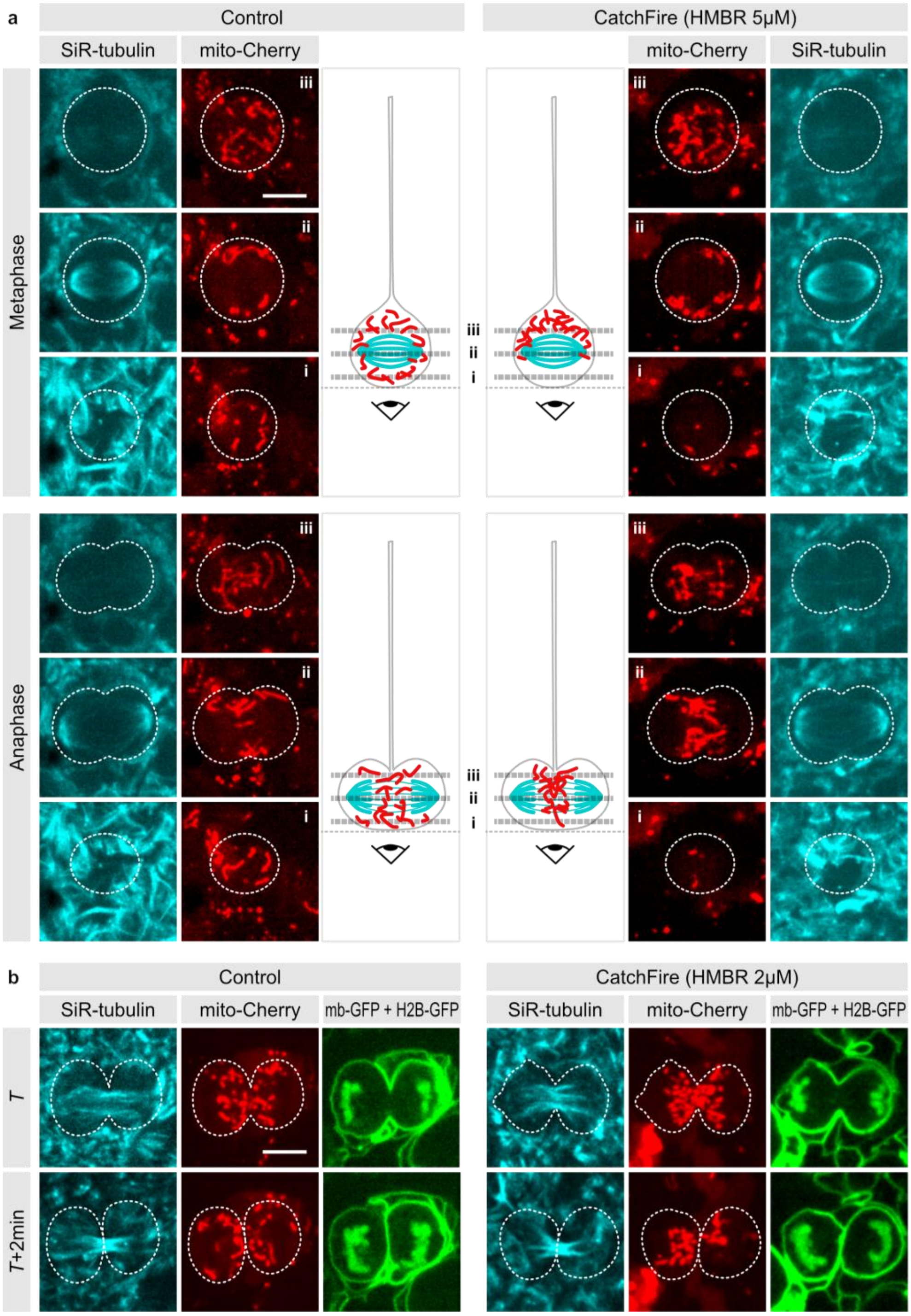

Supplementary Fig.5. Mitochondrial behavior and localization during metaphase and anaphase are modified in CatchFire electroporated cells upon ligand (HMBR) administration. Related to Fig.3.

a. z-projections (2.5μm corresponding to 5 z-levels) at three different levels (top, middle and bottom) of dividing progenitors, (see schematic representation in the middle panels) during metaphase, (upper panels) and anaphase (lower panels). Mitochondria (mito-Cherry, red), which are typically dispersed throughout the cytoplasm of the mitotic cell in metaphase in

control conditions (left panels), are located more basally in ligand exposure conditions (right panels). In anaphase, mitochondria appeared more concentrated at the cleavage furrow upon ligand administration (right panels) compared to the control situation (left panels). The microtubule network (cyan) is labelled with SiR-tubulin. Scale bars: 5µm

b. z-projections (1.5µm corresponding to 3 z-levels) at the level of the central spindle of dividing progenitors at two successive timepoints (2 min interval) around cytokinesis, in CatchFire electroporated cells without (left) or in presence of the ligand (right). Mitochondria (mito-Cherry, red) are more clustered at the cleavage plane in presence of the ligand. The microtubule network (SiR-tubulin, cyan) does not appear different between the two conditions. The cell contour and chromosomes (green) are revealed by membrane-GFP (mbGFP) and Histone-GFP (H2B-GFP) expression, respectively. Scale bars: 5µm

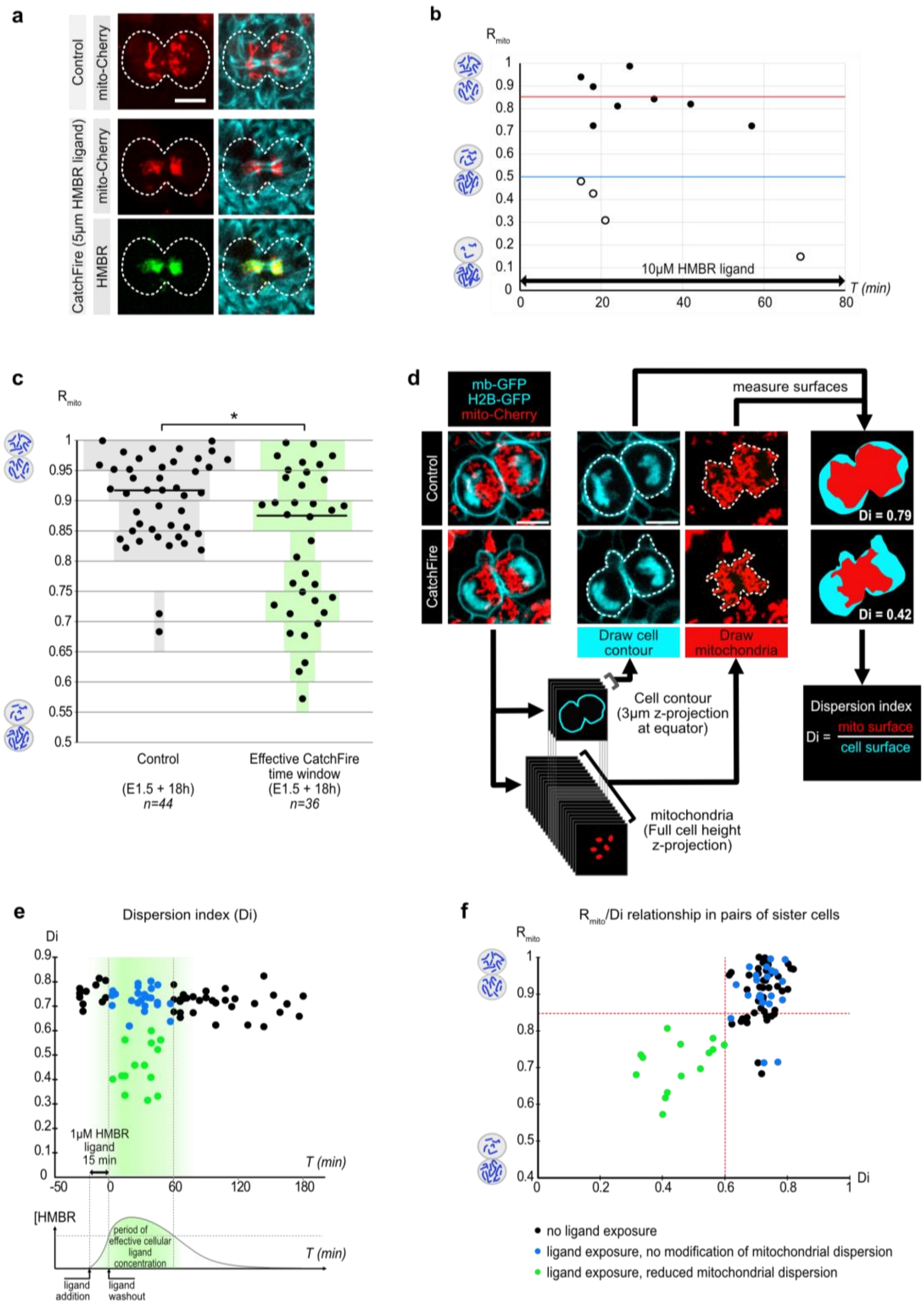

Supplementary Fig.6. CatchFire-induced mitochondria/Kif17 interactions modify mitochondrial behavior and localization. Related to Fig.3.

a. Visualization of the sites of mitochondria/Kif17 interaction in telophase. The interaction between the two partners (Tom20-FireMate, Kif17-FireTag) in the presence of the HMBR ligand is visualized via green fluorescence (HMBR, green) (see Fig.3A), which colocalizes with an independent mitochondrial reporter (mito-Cherry, yellow). The signal also

partially colocalizes with the microtubular network (SiR-tubulin, cyan). Mitochondria are more concentrated near the cleavage furrow in ligand exposure conditions (middle and lower panels) compared to control condition (upper panels). Scale bars: 5µm.

b. Graphical representation of mitochondrial ratios over time during continuous exposure to the HMBR ligand at high concentration (10µM). Each dot represents a mitochondrial ratio. The red line shows the threshold (0.85) between "equal" and "unequal" R_{mito} values and the blue line shows the lower limit (0.5) of R_{mito} observed in control conditions. Open circles show cases of R_{mito} lower than 0.5, indicating an extreme imbalance in mitochondrial inheritance that is never observed in control conditions. The ligand was added to the culture medium at T=0min and maintained in the culture during the entire experimental period. N=12 cells from 1 embryo.

c. Scatter plots of R_{mito} in CatchFire condition before and after (Control) and during the estimated 1 hour period of effective intracellular CatchFire ligand concentration defined in Fig.3B. Gray and green color bars represent the percentage of cell pairs in each 0.05 interval. Data are the same as in Fig.3B. Statistical analysis: Two-tailed Mann-Whitney test. *: p=0.0137 n=80 pairs from 4 embryos.

d. Schematic representation of the measurement of the Dispersion index (Di), illustrated by live imaging of dividing progenitors (upper panels). To determine Di, we used a z-projection of the total mitochondrial signal from the whole cellular volume (mito-Cherry, red) and a z-projection of the maximum cell contour (3 to 5 z-levels at the cell's equator; mb-GFP, green) at the time of cytokinesis. The contours of the projected cell and mitochondrial surfaces were manually drawn (middle panel, green and red respectively). Di is calculated as the ratio of the corresponding surfaces (mitochondrial over equatorial cell surface of the daughter cell pairs). Examples of control (top) and HMBR treated (bottom) sister cells are shown. Scale bars: 5µm.

e. Graphical representation of Di in cells electroporated with the CatchFire components, measured before (-50 to -15 min) addition of the ligand, in presence of ligand in the medium (-15 to 0 min), and following ligand removal from the medium (0 to 180 min). Each dot represents Di for 1 pair of sister cells. Many cases of Di values lower than 0.6 (green dots) are observed during a 1 hour time window after ligand removal from the medium (green box), whereas all values are higher than 0.6 before and after this time window. This corresponds to the time period during which we observed low values of R_{mito} in the same experiment (also marked with a green box in Fig.3B). Blue dots represent pairs measured during the period of effective ligand activity which retained a Di similar to the control condition. Bottom: Scheme of the theoretical variation of the intracellular ligand concentration during the experimental period. Data are from the same

dataset as in panel C and in Fig.3B, except that Di could not be measured in one control pair. N=79 pairs from 4 embryos.

f. Graphical representation of the relationship between R_{mito} and Di values measured at E2.25 in cells electroporated at E1.5 with the CatchFire components, corresponding to the experimental dataset depicted in panels C and E and in Fig.3B. Blue and green dots correspond to cells dividing during the 1 hour time window highlighted by the green box in panel E and Fig.3B. Note that in this population, all the green cells with a Di value lower than 0.6 (which is never observed in the control population) display a R_{mito} lower than 0.85, whereas most of the blue dots have similar R_{mito} as the control population. N=79 pairs from 4 embryos.

E = Embryonic day; h = hours; min = minutes. Source data are provided as a Source Data file. Green shading in panel e represents the deduced period of ligand effect.

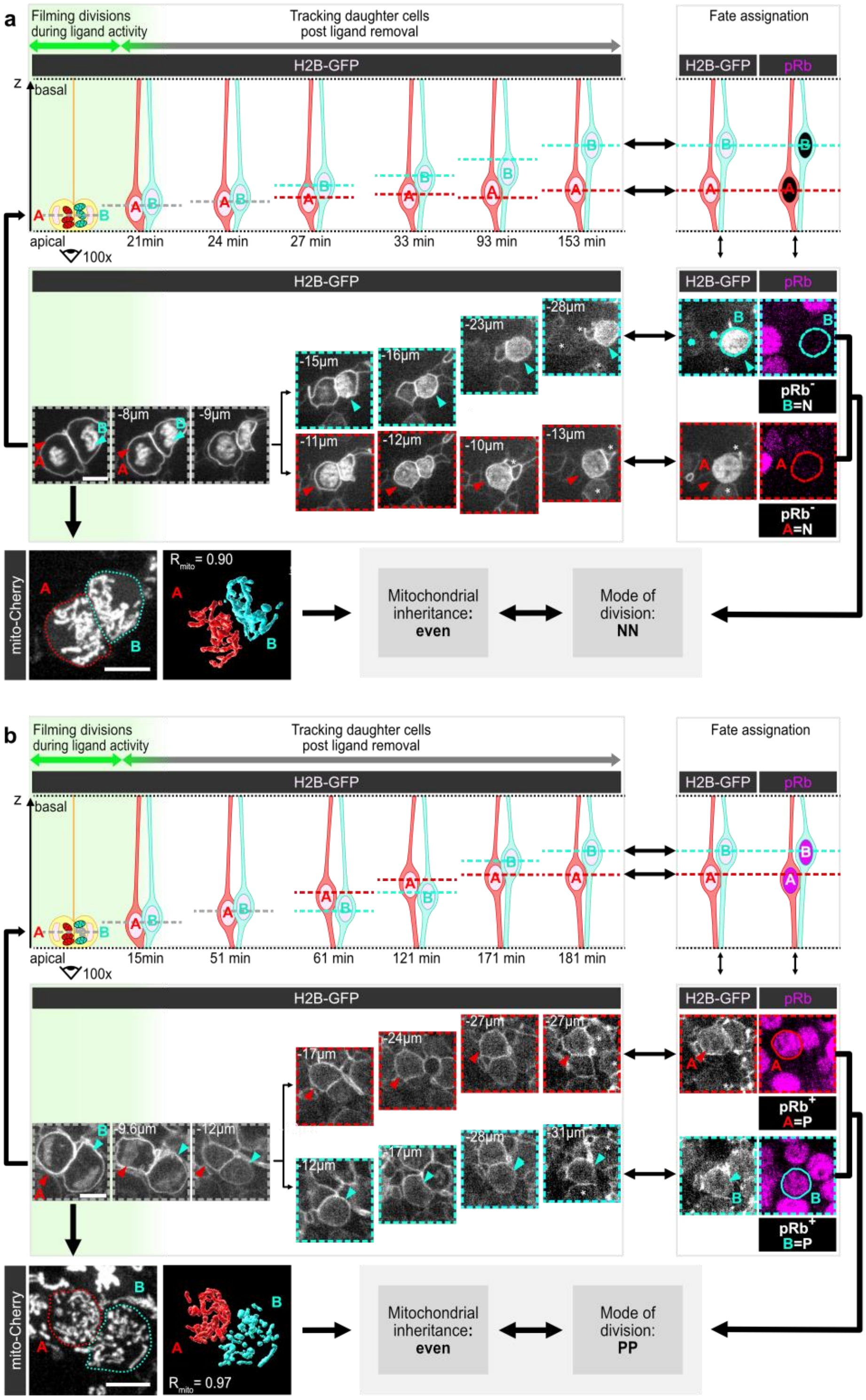

Supplementary Fig.7. Forced unequal mitochondrial inheritance induces asymmetric fate choices. Related to Fig.3.

Representative examples of a NN division **(a)** and a PP division **(b)** both displaying a characteristic equal mitochondrial inheritance.

Scheme (top) and confocal en-face views (middle) of daughter cell's nucleus (H2B-GFP, white) in the depth of the neuroepithelium during the live tracking period (left) and correspondence with pRb immunofluorescence at its end (right, magenta).

Images corresponding to z-levels of the two sister cells are color coded (dotted frame) to illustrate the correspondence with the schematics of the time course.

Bottom: R_{mito} value measured upon cytokinesis of the mother cell is matched with daughter cells fate deduced from tracking: Scale bar: 5µm.

P = progenitor; N = neuron; min = minutes. Green shading represents the deduced period of ligand effect.

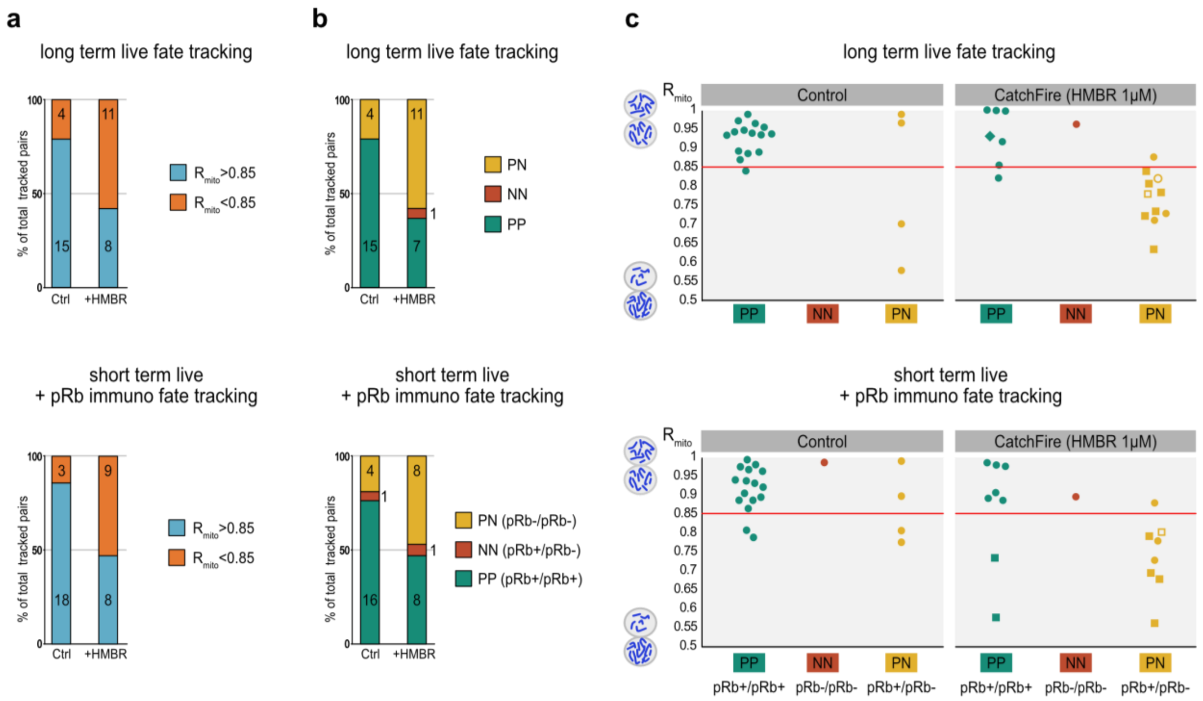

Supplementary Fig.8. Forced unequal mitochondrial segregation in progenitors dividing mostly symmetrically leads to differential cell fate in daughter cells. Related to Fig.3.

a. Graphical representation of percentages of divisions displaying R_{mito} values higher versus lower than 0.85 in the live tracking experiments in control and CatchFire conditions, from the long-term live tracking (top) and from the combined short tracking and immunostaining (bottom) experiments. Numbers indicated in columns correspond to cell numbers. Data are from the same experiments presented in a combined format in Fig.3E.

b. Graphical representation of percentages of PP, PN and NN pairs of sister cells in control and CatchFire conditions in the live tracking experiments, from the long-term live tracking (top) and from the combined short tracking and immunostaining (bottom) experiments. Numbers indicated in columns correspond to cell numbers. Data are from the same experiments presented in a combined format in Fig.3F.

c. Correspondence between mitochondrial inheritance and fate of daughter cells in control and CatchFire conditions. The datasets obtained from the two independent methods for fate assignation based either on long term live tracking (n=19 control cells and n=19 cells in CatchFire condition) or on combined short tracking and immunostaining (n=21 control cells and n=17 cells in CatchFire condition) are presented in the upper and lower rows respectively. They are from the same experiments presented in a combined format in Fig.3G.

For long term live fate tracking, n=2 embryos for control and n=2 embryos for CatchFire pairs. For short term live + pRb immuno fate tracking, n=2 embryos for control and n=2 embryos for CatchFire pairs.

P = progenitor; N = neuron. Source data are provided as a Source Data file.

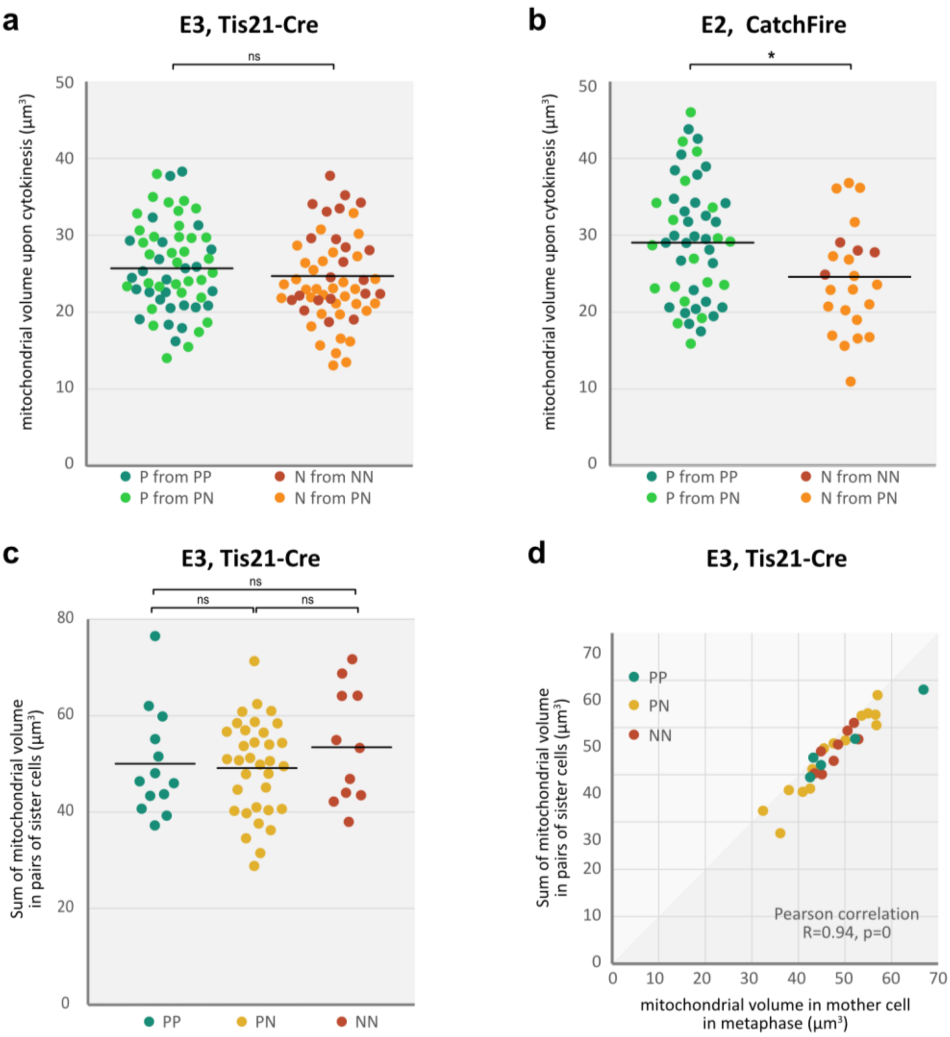

Supplementary Fig.9: Overlapping distribution of absolute mitochondrial volume between progenitors and neurons.

a. Graph plotting the inherited mitochondrial volume inherited upon cytokinesis by cells that later adopt a progenitor (red and orange) versus neuronal (yellow and green) fate in the Tis21-Cre progenitor population at E3 (right). Data are from 58 tracked pairs of cells whose R_{mito} and fate are presented in Fig.2 (Tis21-Cre, E3). n=60 P (26 born from PP and 34 born from PN divisions) and n=56 N (34 born from PN and 22 born from NN divisions) from 5 embryos. Black lines: mean value of distribution. Two-tailed unpaired students t-test: ns

b. Graph plotting the inherited mitochondrial volume inherited upon cytokinesis by cells that later adopt a progenitor (red and orange) versus neuronal (yellow and green) fate upon ligand exposure in CatchFire experiments at E2. Data are from tracked cells whose R_{mito} and fate are presented in Fig.3. n=49 P (30 born from PP and 19 born from PN divisions) and n=23

N (19 born from PN and 4 born from NN divisions) from 4 embryos. Black lines: mean value of distribution. Two-tailed unpaired students t-test: p=0.01.

c. Graph plotting the mitochondrial volume of dividing Tis21-Cre progenitors at E3, categorized as PP (red), PN (yellow) and NN (green) according to the fate of their progeny. The sum of the mitochondrial volume of daughter cells was used as a proxy to evaluate the volume in their mother cell, as validated in panel C. Data are from 58 tracked pairs of cells from 5 embryos whose R_{mito} and fate are presented in Fig.2 (Tis21-Cre, E3). PP: n=13; PN: n=34; NN: n=11. Black lines: mean value of distribution. Two-tailed unpaired students t-test: ns

d. Graph plotting the sum of the inherited mitochondrial volume in daughter cells (Y axis) and the measured mitochondrial volume of the corresponding mother cell in metaphase (X axis) in a subset of tracked Tis21-Cre cells at E3. The data show equality between the sum of the volume inherited by daughter cells and the volume in their mother in metaphase. Pearson correlation coefficient analysis revealed a significant correlation between the two variables. PP: n=5; PN: n=15; NN: n=8 for progenitors and corresponding pairs of daughters.

E = Embryonic day; P = progenitor; N = neuron. Source data are provided as a Source Data file.

Supplementary Table I: Key resources

REAGENT OR RESSOURCE	SOURCE	IDENTIFIER
**Antibodies**		
Chick anti-GFP	Aves Labs	Cat#GFP-1020<br>RRID : AB_10000240
Rabbit anti-pRb (Ser807/811)	Cell Signaling	Cat# 8516S<br>RRID : AB_331472
Mouse anti-HuC/D (clone 16A11)	Thermo Fisher Scientific	Cat#A-21271<br>RRID : AB_221448
**Softwares**		
ImageJ		http://imagej.net/Welcome<br>RRID: SCR_003070
Graphpad Prism 6.0	Graphpad	http://www.graphpad.com/<br>RRID: SCR_002798
Microsoft Excel	Microsoft	RRID: SCR_016137
MicroManager		https://micro-manager.org/<br>RRID: SCR_000415
Affinity Publisher 2.6	Affinity	N/A
IMARIS 10.0.0	BitPlane	N/A

Plasmids

Name	SOURCE	IDENTIFIER
pCX-mbGFP-ires-H2BGFP (X-411; membrane-GFP + Histone2B-GFP)	This study	N/A
pCX-mbiRFP670 (X-713 ; membrane iRFP670)	This study	N/A
pCX-ZO1-miRFP670 (X-786 ; miRFP670-ZO1)<br>ZO1 iRFP670	This study	N/A
pCX-H2B-EGFP (X-014; Histone2B GFP	Morin et al, 2007	N/A
PMP-Cdc25b (X-521; Human Cdc25b3 under control of the pccRE element of the mouse Cdc25b promoter)	Bonnet et al, 2018	N/A

PMP-lacZ (X-1090; Control vector expressing lacZ under control of the pccRE element of the mouse Cdc25b promoter)	Bonnet et al, 2018	N/A
Cdkn1c SH1 (X-1101; Chick Cdkn1c miRNA under control of chick U6 promoter, H2B-EGFP under control of CAGGS promoter)	Mida et al, 2024	N/A
shCTRL (KD-048; Control miRNA targeting the luciferase sequence under control of chick U6 promoter, H2B-EGFP under control of CAGGS promoter)	Morin et al, 2007	N/A
Cytobow (X-400; cytoplasmic Brainbow vector)	Loulier et al, 2014	N/A
MitoBow (X-503; mitochondrial Brainbow vector)	Loulier et al, 2014	N/A
pCX-Mito-CFP (X-508; mECFP-tagged mitochondrial reporter)	Unpublished, gift from Jean Livet	N/A
pCX-lox-H2B-EBFP-lox-mitoEGFP (X-1017; Cre-recombinase dependent expression of EGFP-tagged mitochondrial)	This study	N/A
pCX-lox-mito-Cherry-lox-mitoEGFP (X-1018; Constitutive expression of a mCherry tagged mitochondrial reporter and Cre-recombinase dependent expression of EGFP-tagged mitochondrial reporter)	This study	N/A
pCX-mito-Cherry (X-1202; mCherry-tagged mitochondrial reporter)	This study	N/A
pCX-Cre (X-020; Cre recombinase under control of the CAGGS promoter)	Morin et al, 2007	N/A
pCX-Tom20-NpFAST (X-1273; TOM20-NpFAST, MitoFireMate)	This study	N/A
pCX-Kif17a-CpFAST (X-1274; Kif17a-CpFAST, KifFireTag)	This study	N/A
cTis21-p2A-NLSCre (X-907; donor vector for somatic knock-in of Cre recombinase in the chick Tis21 locus)	Petit-Vargas et al, 2024	N/A

pCX-SpCas9-cTis21-gRNA2 (X-854; Expresses a gRNA targeting the C-terminus of the chick Tis21 locus under control of human U6 promoter, and the SpCas9 nuclease under control of the hybrid CMV-Chicken b-actin promoter)	Petit-Vargas et al, 2024	N/A

Supplementary references

Bonnet, F. et al. Neurogenic decisions require a cell cycle independent function of the CDC25B phosphatase. Elife 3;7:e32937, (2018).

Loulier, K. et al. Multiplex Cell and Lineage Tracking with Combinatorial Labels. Neuron 81, 505–520 (2014).

Mida, B. et al. A low Cdkn1c/p57kip2 expression in spinal progenitors drives the transition from proliferative to neurogenic modes of division. Preprint at https://doi.org/10.1101/2024.10.10.617342 (2024).

Morin, X., Jaouen, F. & Durbec, P. Control of planar divisions by the G-protein regulator LGN maintains progenitors in the chick neuroepithelium. Nat Neurosci 10, 1440–1448 (2007).

Petit-Vargas, A. P. et al. CRISPR/Cas9-based somatic knock-in of reporters in the avian embryo in ovo. Preprint at https://doi.org/10.1101/2024.10.10.617291 (2024).
