# Supplementary material for: Unequal mitochondrial segregation promotes asymmetric fates during neurogenesis
Source: Nat Commun. 2025 Dec 15;16:11049. doi: 10.1038/s41467-025-66932-0 (PMC12706016; doi:10.1038/s41467-025-66932-0)
Supplement: Supplementary file 3 — Description of Additional Supplementary Files [file 41467_2025_66932_MOESM3_ESM.pdf]

Description of Additional Supplementary Files

File Name: Supplementary Movie 1
Description: En-face time lapse monitoring of mitochondrial segregation during progenitor cellular divisions in chick embryonic neuroepithelium. Related to Fig.1. Plasmids encoding mito-Cherry (red) and mb-iRFP (cyan) were electroporated in the neural tube in ovo, at embryonic day 2 (E2, HH stage 13–14). 24 hours later, embryos were dissected, and the neuroepithelium was imaged in en-face view every 3minutes during one hour. The movie displays a single focal plane from a z-stack, 5µm below the apical surface. White rectangles indicate pairs of sister cells immediately after cytokinesis, corresponding to the time points used for 3D reconstructions and measurements of mitochondrial volume inherited by each sister in this study. Scale bar, 20µm.

File Name: Supplementary Movie 2
Description: Mitochondrial segregation during cellular division of a neural progenitor in the chick embryonic neuroepithelium. Related to Fig.1 and Fig. S1. Plasmids encoding mito-Cherry (red) and a mb-GFP-ires-H2B-GFP (cyan) were electroporated in the neural tube in ovo, at embryonic day 2 (E2, HH stage 13–14) and the neuroepithelium was imaged in en-face view 24 hours later. Upper row of the movie: single focal planes at 4 consecutive timepoints from metaphase to cytokinesis of a single progenitor, 6 µm below the apical surface. Bottom row shows sequential views of all single z-planes within the z-stack in metaphase (left) and after cytokinesis (right). The latter was imported in Imaris for 3D-reconstruction of mitochondrial and cellular volumes in the two daughter cells (middle). In this example, both daughter cells inherit similar mitochondrial volumes from the mother cell (Rmito=0.97). Scale bar in confocal images, 5µm.

File Name: Supplementary Movie 3
Description: Combined monitoring of mitochondrial inheritance and sister cell fate in an asymmetrically dividing progenitor (PN). Related to Fig.2. Plasmids encoding fluorescent reporters for mitochondria (lox-mito-GFP), apical tracking (iRFP-ZO1), cytoplasm staining (Cytobow, used to identify the cellular contour for 3D reconstruction; not shown in the movie) and constructs for the targeting of the Cre recombinase at the Tis21 locus were electroporated in the neural tube at embryonic day 2 and the neuroepithelium was imaged in en-face view 24 hours later. The left panel shows the 3D reconstruction of mitochondrial volumes inherited by each sister A and B, indicating unbalanced inheritance (Rmito = 0.61) with B receiving a smaller volume. The right panel shows a time-lapse series of the apical iRFP-ZO1 signal used for long term tracking of pairs of daughter cells. Daughter cell B undergoes a progressive reduction of its apical surface followed by delamination at 24 hours and is therefore identified as a neuron, while daughter cell A divides at 26 hours and is therefore identified as a progenitor. Note that the daughter cell inheriting the smallest mitochondrial volume becomes a neuron.

File Name: Supplementary Movie 4
Description: Combined monitoring of mitochondrial inheritance and sister cell fate in a symmetric proliferative progenitor division (PP). Related to Fig.2 and Fig. S4. Plasmids encoding fluorescent reporters for mitochondria (lox-mito-GFP), apical tracking (iRFP-ZO1) and cytoplasm staining (Cytobow, used to identify the cellular contour for 3D reconstruction; not shown in the movie) and constructs for the targeting of the Cre recombinase at the Tis21 locus were electroporated in the neural tube at embryonic day 2 and the neuroepithelium was imaged in en-face view 24 hours later. The left panel shows the 3D reconstruction of mitochondrial volumes inherited by each sister A and B, indicating balanced inheritance (Rmito = 0.92). The right panel shows a time-lapse series of the apical iRFP-ZO1

signal used for long term tracking of pairs of daughter cells. Both daughter cells undergo a new round of division at 22 hours (A) and 26 hours (B) and are therefore identified as progenitors

File Name: Supplementary Movie 5
Description: Combined monitoring of mitochondrial inheritance and sister cell fate in a symmetric neurogenic progenitor division (NN). Related to Fig.2 and Fig. S4. Plasmids encoding fluorescent reporters for mitochondria (lox-mito-GFP), apical tracking (iRFP-ZO1) and cytoplasm staining (Cytobow, used to identify the cellular contour for 3D reconstruction; not shown in the movie) and constructs for the targeting of the Cre recombinase at the Tis21 locus were electroporated in the neural tube at embryonic day 2 and the neuroepithelium was imaged in en-face view 24 hours later. The left panel shows the 3D reconstruction of mitochondrial volumes inherited by each sister A and B, indicating balanced inheritance (Rmito = 0.97). The right panel shows a time-lapse series of the apical iRFP-ZO1 signal used for long term tracking of pairs of daughter cells. Both daughter cells undergo a progressive reduction of their apical surface followed by delamination at 24 hours (B) and 30 hours (A) and are therefore identified as neurons.

File Name: Supplementary Movie 6
Description: Mitochondrial localization is modified during metaphase in CatchFire electroporated cells upon ligand (HMBR) administration. Related to Fig.3 and Supplementary Fig.5. The movie presents an animated 3D-reconstruction in Imaris of mitochondrial and cellular volumes in metaphase cells. Mitochondria (mito-Cherry, red), which are typically dispersed throughout the cytoplasm in control conditions (left cell), are located more basally upon ligand exposure (right cell).

File Name: Supplementary Movie 7
Description: Mitochondrial localization is modified during anaphase in CatchFire electroporated cells upon ligand (HMBR) administration. Related to Fig.3 and Supplementary Fig.5. The movie presents an animated 3D-reconstruction in Imaris of mitochondrial and cellular volumes in anaphase cells. Mitochondria (mito-Cherry, red) appeared more concentrated at the cleavage furrow upon ligand administration (right panels) compared to the control situation (left panels).

File Name: Supplementary Movie 8
Description: dentification of a “PN” pair of sister cells using a combination of live tracking after cytokinesis and pRb immunofluorescence. Related to Fig.3. Sister cells expressing mb-GFP and H2B-GFP, CatchFire components and a mitochondrial reporter (mito-Cherry) were imaged in en-face cultures of the neuroepithelium at E2.25. z-stacks were acquired at 3 minutes intervals and 0.3µm z steps for 1 hour, then at 15 minutes intervals and 1µm z steps for an additional 2.5 hours. The two left columns show single focal planes from the live sequence. Only the GFP channel is shown. Due to interkinetic nuclear movements, both sisters’ nuclei move in the depth of the neuroepithelium at different speeds, and are therefore displayed on two different rows (top and bottom) from 45 minutes onwards until the end of the live imaging sequence. Samples were fixed at the end of the live imaging sequence and processed for immunostaining with anti-GFP and pRb antibodies. The third column shows the correspondence of the anti-GFP signal in the fixed sample with the last time point of the live tracking sequence, and the 4th column shows the pRb signal. Cell A (top row) is pRb negative, and therefore identified as a neuron, while cell B (bottom row) is pRb positive and identified as a cycling progenitor. T=0 minute corresponds to the timing of cytokinesis of the mother cell. Asterisks indicate neighboring cells used for registration between live and fixed images.

File Name: Supplementary Movie 9

Description: Identification of a “PP” pair of sister cells using a combination of live tracking after cytokinesis and pRb immunofluorescence. Related to Fig.3 and Fig. S7. Sister cells expressing mb-GFP and H2B-GFP, CatchFire components and a mitochondrial reporter (mito-Cherry) were imaged in en-face cultures of the neuroepithelium at E2.25. z-stacks were acquired at 3 minutes intervals and 0.3µm z steps for 1 hour, then at 10 minutes intervals and 1µm z steps for an additional 2 hours. The two left columns show single focal planes from the live sequence. Only the GFP channel is shown. Due to interkinetic nuclear movements, both sisters’ nuclei move in the depth of the neuroepithelium at different speeds, and are therefore displayed on two different rows (top and bottom) from 49 minutes onwards until the end of the live imaging sequence. Samples were fixed at the end of the live imaging sequence and processed for immunostaining with anti-GFP and pRb antibodies. The third column shows the correspondence of the anti-GFP signal in the fixed sample with the last time point of the live tracking sequence, and the 4th column shows the pRb signal. Both cell A (top row) and cell B (bottom row) are pRb positive and identified as cycling progenitors. T=0 minute corresponds to the timing of cytokinesis of the mother cell. Asterisks indicate neighboring cells used for registration between live and fixed images.

File Name: Supplementary Movie 10
Description: dentification of a “NN” pair of sister cells using a combination of live tracking after cytokinesis and pRb immunofluorescence. Related to Fig.3 and Fig. S7. Sister cells expressing mb-GFP and H2B-GFP, CatchFire components and a mitochondrial reporter (mito-Cherry) were imaged in en-face cultures of the neuroepithelium at E2.25. z-stacks were acquired at 3 minutes intervals and 0.3µm z steps for 1 hour, then at 10 minutes intervals and 1µm z steps for an additional 2 hours. The two left columns show single focal planes from the live sequence. Only the GFP channel is shown. Due to interkinetic nuclear movements, both sisters’ nuclei move in the depth of the neuroepithelium at different speeds, and are therefore displayed on two different rows (top and bottom) from 45 minutes onwards until the end of the live imaging sequence. Samples were fixed at the end of the live imaging sequence and processed for immunostaining with anti-GFP and pRb antibodies. The third column shows the correspondence of the anti-GFP signal in the fixed sample with the last time point of the live tracking sequence, and the 4th column shows the pRb signal. Both cell A (top row) and cell B (bottom row) are pRb negative, and therefore identified as neurons. T=0 minute corresponds to the timing of cytokinesis of the mother cell. Asterisks indicate neighboring cells used for registration between live and fixed images.
