# Supplementary material for: Unequal mitochondrial segregation promotes asymmetric fates during neurogenesis
Source: Nat Commun. 2025 Dec 15;16:11049. doi: 10.1038/s41467-025-66932-0 (PMC12706016; doi:10.1038/s41467-025-66932-0)
Supplement: Supplementary file 14 — Reporting Summary [file 41467_2025_66932_MOESM14_ESM.pdf]

nature portfolio

Corresponding author(s): Evelyne FISCHER and Xavier MORIN

Last updated by author(s): 2025/11/18

Reporting Summary

Nature Portfolio wishes to improve the reproducibility of the work that we publish. This form provides structure for consistency and transparency in reporting. For further information on Nature Portfolio policies, see our Editorial Policies and the Editorial Policy Checklist.

Statistics

For all statistical analyses, confirm that the following items are present in the figure legend, table legend, main text, or Methods section.

n/a	Confirmed	
☐	☒	The exact sample size (*n*) for each experimental group/condition, given as a discrete number and unit of measurement
☐	☒	A statement on whether measurements were taken from distinct samples or whether the same sample was measured repeatedly
☐	☒	The statistical test(s) used AND whether they are one- or two-sided<br>*Only common tests should be described solely by name; describe more complex techniques in the Methods section.*
☒	☐	A description of all covariates tested
☒	☐	A description of any assumptions or corrections, such as tests of normality and adjustment for multiple comparisons
☐	☒	A full description of the statistical parameters including central tendency (e.g. means) or other basic estimates (e.g. regression coefficient) AND variation (e.g. standard deviation) or associated estimates of uncertainty (e.g. confidence intervals)
☐	☒	For null hypothesis testing, the test statistic (e.g. *F*, *t*, *r*) with confidence intervals, effect sizes, degrees of freedom and *P* value noted<br>*Give P values as exact values whenever suitable.*
☒	☐	For Bayesian analysis, information on the choice of priors and Markov chain Monte Carlo settings
☒	☐	For hierarchical and complex designs, identification of the appropriate level for tests and full reporting of outcomes
☒	☐	Estimates of effect sizes (e.g. Cohen's *d*, Pearson's *r*), indicating how they were calculated

Our web collection on statistics for biologists contains articles on many of the points above.

Software and code

Policy information about availability of computer code

Data collection	Image acquisition: MicroManager, https://micro-manager.org/ RRID: SCR_000415
Data analysis	Graphpad Prism 6 for statistical analyses (http://www.graphpad.com/ RRID: SCR_002798)<br>Microsoft Excel, Microsoft RRID: SCR_016137<br>IMARIS 10.0.0, BitPlane

For manuscripts utilizing custom algorithms or software that are central to the research but not yet described in published literature, software must be made available to editors and reviewers. We strongly encourage code deposition in a community repository (e.g. GitHub). See the Nature Portfolio guidelines for submitting code & software for further information.

Data

Policy information about availability of data

All manuscripts must include a data availability statement. This statement should provide the following information, where applicable:

- Accession codes, unique identifiers, or web links for publicly available datasets
- A description of any restrictions on data availability
- For clinical datasets or third party data, please ensure that the statement adheres to our policy

Source data are provided with this paper. Plasmids used in this study and their full sequences are available upon request to X.M.

Research involving human participants, their data, or biological material

Policy information about studies with human participants or human data. See also policy information about sex, gender (identity/presentation), and sexual orientation and race, ethnicity and racism.

Reporting on sex and gender	NA
Reporting on race, ethnicity, or other socially relevant groupings	NA
Population characteristics	NA
Recruitment	NA
Ethics oversight	NA

Note that full information on the approval of the study protocol must also be provided in the manuscript.

Field-specific reporting

Please select the one below that is the best fit for your research. If you are not sure, read the appropriate sections before making your selection.

[X] Life sciences [ ] Behavioural & social sciences [ ] Ecological, evolutionary & environmental sciences

For a reference copy of the document with all sections, see nature.com/documents/nr-reporting-summary-flat.pdf

Life sciences study design

All studies must disclose on these points even when the disclosure is negative.

Sample size	We did not perform any a priori samples size calculation. A first round of experiments at different embryonic stages was performed measuring more than 50 mitotic events in total from at least three embryos. The analysis of these initial datasets showed that this sample size was sufficient to determine significant differences between conditions and to support meaningful conclusions. This sample size was used as a reference for subsequent experiments, with the exception of two experiments (Figure 2A and Figure 3E-G) for which a slightly smaller sample size was used.
Data exclusions	No data were excluded post measurement.
Replication	All measurements of Rmito, Rcell, Di, Vmito and Vcell values are pooled from at least three independent live imaging experiments performed on single embryos, since our live imaging experiments can be performed in only one embryo at a time. The number of embryos for each experimental condition is indicated in the figure legends. All attempts at replication were successful.
Randomization	Randomisation was not relevant to this study: experimental groups were defined on the basis of the experimental treatment (e.g., combination of electroporated vectors and/or treatment with the CatchFire ligand), and relevant controls were performed and reported for each treatment.
Blinding	For all measurements of Rmito, blinding between experimental conditions and their control was not relevant: the measurement for any individual pair of cells is not predictable a priori by eye, and variables were quantified using standardized means (Imaris). Of note, for “tracked” experiments (Figure 2e-g), the fate of sister cells was allocated before Rmito measurements and blinded for the experimenter doing the ratio measurement.

Reporting for specific materials, systems and methods

We require information from authors about some types of materials, experimental systems and methods used in many studies. Here, indicate whether each material, system or method listed is relevant to your study. If you are not sure if a list item applies to your research, read the appropriate section before selecting a response.

Materials & experimental systems

n/a	Involved in the study	
☐	☒	Antibodies
☒	☐	Eukaryotic cell lines
☒	☐	Palaeontology and archaeology
☐	☒	Animals and other organisms
☒	☐	Clinical data
☒	☐	Dual use research of concern
☒	☐	Plants

Methods

n/a	Involved in the study	
☒	☐	ChIP-seq
☒	☐	Flow cytometry
☒	☐	MRI-based neuroimaging

Antibodies

Antibodies used	Primary antibodies used are:<br>Chick anti-GFP, Aves Labs Cat# GFP-1020 RRID : AB_10000240, dilution 1:800.<br>Rabbit anti-pRb (Ser807/811), Cell Signaling, Cat# 8516S RRID : AB_331472, dilution 1:1000.<br>Mouse anti-HuC/D (clone 16A11), Thermo Fisher Scientific, Cat# A-21271 RRID : AB_221448, dilution 1:50
Validation	These commercial antibodies have been validated in prior studies in our model (Baek et al, 2018; Molina et al 2022; Tozer et al, 2017)

Animals and other research organisms

Policy information about studies involving animals; ARRIVE guidelines recommended for reporting animal research, and Sex and Gender in Research

Laboratory animals	JA57 chicken fertilized eggs were provided by EARL Morizeau (8 rue du Moulin, 28190 Dangers, France). Eggs were incubated and manipulated between embryonic days 1.5 and 4.
Wild animals	NA
Reporting on sex	The sex of the embryos was not determined.
Field-collected samples	NA
Ethics oversight	Under current European Union regulations, experiments on avian embryos between 2 and 4 days in ovo are not subject to restrictions.

Note that full information on the approval of the study protocol must also be provided in the manuscript.

Plants

Seed stocks	NA
Novel plant genotypes	NA
Authentication	NA
